# Intra-lymphatic administration of GAD-alum in type 1 diabetes: long-term follow-up and effect of a late booster dose (the DIAGNODE Extension trial)

**DOI:** 10.1007/s00592-022-01852-9

**Published:** 2022-01-31

**Authors:** Rosaura Casas, Fabrícia Dietrich, Sara Puente-Marin, Hugo Barcenilla, Beatriz Tavira, Jeannette Wahlberg, Peter Achenbach, Johnny Ludvigsson

**Affiliations:** 1 Division of Pediatrics, Department of Biomedical and Clinical Sciences, Faculty of Medicine and Health Sciences, Linköping University, Linköping, Sweden; 2 Department of Endocrinology and Department of Medical and Health Sciences and Department of Clinical and Experimental Medicine, Linköping University, Linköping, Sweden; 3 Institute of Diabetes Research, School of Medicine, Forschergruppe Diabetes, Helmholtz Zentrum München, Technical University of Munich, Munich, Germany; 4 Division of Pediatrics, Department of Biomedical and Clinical Sciences, Faculty of Medicine Health Sciences and Crown Princess, Victoria Children’s Hospital, Linköping University, 58185 Linköping, SE Sweden

**Keywords:** Autoantigen, Immunotherapy, GAD-alum, Intra-lymphatic, Type 1 diabetes, Booster dose

## Abstract

**Aim:**

To evaluate the long-term effect of intra-lymphatic administration of GAD-alum and a booster dose 2.5 years after the first intervention (DIAGNODE Extension study) in patients with recent-onset type 1 diabetes.

**Methods:**

DIAGNODE-1: Samples were collected from 12 patients after 30 months who had received 3 injections of 4 μg GAD-alum into a lymph node with one-month interval. DIAGNODE Extension study: First in human, a fourth booster dose of autoantigen (GAD-alum) was given to 3 patients at 31.5 months, who were followed for another 12 months. C-peptide was measured during mixed meal tolerance tests (MMTTs). GADA, IA-2A, GADA subclasses, GAD_65_-induced cytokines, PBMCs proliferation and T cells markers were analyzed.

**Results:**

After 30-month treatment, efficacy was still seen in 8/12 patients (good responders, GR). Partial remission (IDAA1c < 9) had decreased compared to 15 months, but did not differ from baseline, and HbA1c remained stable. GAD_65_-specific immune responses induced by the treatment started to wane after 30 months, and most changes observed at 15 months were undetectable. GADA subclasses IgG2, IgG3 and IgG4 were predominant in the GR along with IgG1. A fourth intra-lymphatic GAD-alum dose to three patients after 31.5 months gave no adverse events. In all three patients, C-peptide seemed to increase the first 6 months, and thereafter, C-peptide, HbA1c, insulin requirement and IDAA1c remained stable.

**Conclusion:**

The effect of intra-lymphatic injections of GAD-alum had decreased after 30 months. Good responders showed a specific immune response. Administration of a fourth booster dose after 31.5 months was safe, and there was no decline in C-peptide observed during the 12-month follow-up.

**Supplementary Information:**

The online version contains supplementary material available at 10.1007/s00592-022-01852-9.

## Introduction

Despite the use of modern devices and drugs, treatment of type 1 diabetes is heavy and the disease cause complications and increased mortality [1, 2]. Immune interventions to preserve residual beta cell function have been tried for several decades [3], but the effect has been limited and transient, and some have been accompanied by serious risks and adverse events [4–12]. Subcutaneous (sc) administration of glutamic acid decarboxylase (GAD) 65 formulated with aluminum hydroxide (GAD-alum) has shown to be easy and safe, but the clinical results have been inconclusive [13–15], even though a meta-analyses showed very high probability that the treatment is efficacious [16]. The treatment needs to be improved.

It is known that intra-lymphatic administration of antigens can maximize immunogenicity and hence the efficacy of several vaccines [17, 18]. Thus, in an attempt to render the presentation of GAD65 more efficient, GAD-alum was injected into an inguinal lymph node in 12 individuals with recent-onset type 1 diabetes in an open-label clinical trial (DIAGNODE-1), being the first-in-human trial with autoantigen given intra-lymphatic. Results from the 6 and 15-month follow-up showed that the treatment was safe, seemed to preserve C-peptide and induced several immunological reactions (19–21). These encouraging results gave rise to a randomized double-blind placebo-controlled Phase II trial, DIAGNODE-2. When this was ongoing, a meta-analyses of the previous studies with sc GAD-alum treatment showed that the efficacy was mainly restricted to patients with HLDR3DQ2 [22], and therefore, efficacy in this subgroup of patients was added in an amendment of the DIAGNODE-2 protocol. In fact, there was a significant efficacy to preserve C-peptide with associated clinical benefit (increased proportion of patients in so-called partial remission (insulin dose-adjusted A1c; IDAA1c < 9) [23, 24].

Now we ask the question how long term the immunological effect will be after three doses of intra-lymphatic GAD-alum injections, and therefore, a follow-up of DIAGNODE-1 has been conducted 30 months after the treatment was initiated. With the hypothesis that the effect is declining, we had already in the end of DIAGNODE-1 applied for permission to study whether the addition of a late intra-lymphatic booster dose of GAD-alum could prolong the efficacy and immune response. A fourth dose as a booster given 2.5 years after the initial treatment was allowed in three adult patients who had earlier participated in DIAGNODE-1, constituting the DIAGNODE Extension study. We are aware of the very low number of patients investigated, but understand that this is a first-in-human trial when the treatment either might lead to deleterious effects on both beta cell function and immune system, with risk for adverse events, or show to have positive effects without adverse events. The result of this study may have great consequences for future trials with autoantigen therapy.

## Research design and methods

### Study design and participants

In DIAGNODE-1 as described earlier [21], 12 individuals (4 females and 8 males), aged 12–24 years with type 1 diabetes < 6 months from diagnosis, received a primary injection of 4 μg of GAD-alum (Diamyd Medical, Stockholm) into an inguinal lymph node administrated by help of ultrasound technique, followed by two booster injections of 4 μg each with one-month interval. In parallel, patients also received vitamin D (calciferol) in oral solution (2000 U/d) for 4 months, starting 1 month prior to first GAD-alum injection. The participants were eligible if fasting C-peptide ≥ 0.12 nmol/L and GAD_65_ antibody levels (GADA) > 63.2 RU [21].

In the first-in-human DIAGNODE Extension study, the Research Ethics Board and the Swedish Medical Product Agency allowed the inclusion of three pilot adult patients who just ended the DIAGNODE-1 trial. They received a fourth injection of GAD-alum (4 μg) into an inguinal lymph node 1.5 months after the 30-month follow-up of DIAGNODE 1, that is, 31.5 months after the start of their first treatment in DIAGNODE-1, in combination with oral vitamin D intake one month before and one month after the fourth intra-lymphatic GAD-alum injection. The patients were followed with mixed meal tolerance tests (MMTTs) [25] and immunological studies for 12 months (NCT02352974).

### Laboratory test

Laboratory analyses were performed at Linköping University, Sweden. Blood and serum samples from all the DIAGNODE-1 participants were collected at baseline and after 15, 30 months. Additionally, samples from the three patients in DIAGNODE Extension were collected at 31.5, 33.5, 36.5 and 42.5 months. Samples were drawn during the morning hours, and peripheral blood mononuclear cells (PBMCs) were isolated within 24 h using Leucosep (Greiner Bio One) according to the manufacturer’s instructions.

Analysis of serum C-peptide was performed using a solid-phase two-sided enzyme immunoassay (Mercodia, Uppsala). Results for each assay were validated with the inclusion of a Diabetes Antigen Control Human (Low/High) (Mercodia, Uppsala, Sweden). The assay is calibrated against the international reference reagent for C-peptide IRR C-peptide 84/510. Inter- and intra-assay variations were 6.6% and 3.5%, respectively.

### Serum antibodies and IgG subclasses

Serum GAD autoantibodies (GADA) were estimated in duplicate by means of a radio-binding assay, using 35S-labeled recombinant human GAD_65_ (rhGAD_65_) as previously described [26]. Sepharose protein A was used to separate free from antibody-bound labeled GAD_65_.

GADA IgG1, IgG2, IgG3 and IgG4 subclasses were measured by radio-binding assays [27] using IgG subclass-specific biotin-labeled mouse–antihuman monoclonal antibodies bound on streptavidin sepharose high-performance beads (GE Healthcare Life Sciences, Freiburg, Germany) [20]. Results were expressed as delta cpm (IgG subclass-specific cpm—anti-rat IgM cpm) and converted to arbitrary units (AUs) proportional to the GADA IgG subclass-specific delta cpm of a local standard serum.

### Lymphocyte proliferation assay

Proliferative responses were analyzed in PBMCs cultured in triplicates in medium alone (AIM-V medium with β-mercaptoethanol), in the presence of 5 μg/mL rhGAD_65_ (Diamyd Medical, Stockholm, Sweden) or with CD3/CD28 beads (Gibco, Life Technologies AS, Oslo, Norway). After 3 days, cells were incubated with 0.2 µCi of [^3^H] thymidine/well (PerkinElmer) for 18 h and thereafter harvested. Proliferation was expressed as stimulation index (SI), calculated as the mean cpm of cells cultured triplicates in the presence of stimulus divided by the mean cpm of cells with medium alone.

### Cytokine secretion assay

PBMCs were cultured for 7 days in the presence of 5 μg/mL rhGAD_65_ or in medium alone at 37 °C in 5% CO_2_, as previously described [28]. The cytokines IL-2, IL-5, IL-10, IL-13, IL-17, tumor necrosis factor (TNF-*α*) and interferon (IFN-*γ*) were measured in cell culture supernatants using Bio-Plex Pro Cytokine Panel (Bio-Rad, Hercules, CA, USA) according to the manufacturer’s instructions. Data were collected using the Luminex 200 ™ (Luminex xMAP™ Corporation, Austin, TX USA). The antigen-induced cytokine secretion level was calculated by subtracting the spontaneous secretion (i.e. secretion from PBMCs cultured in medium alone) from the one following stimulation with GAD_65_.

### Flow cytometry

PBMCs were washed in PBS containing 0.1% BSA and subsequently stained with Alexa-700-conjugated anti-CD3 (clone UCHT1, BD Biosciences), Pacific Blue-conjugated anti-CD4 (clone RPA-T4, BD Biosciences), allophycocyanin (APC)-H7-conjugated anti-CD8 (clone SK1, BD Biosciences), PerCP-Cy5.5-conjugated anti-CD45RA (clone HI100, BD Biosciences), phycoerythrin (PE)-conjugated anti-CCR7 (clone G043H7, Biolegend), FITC-conjugated anti-CD127 (clone eBioRDR5, eBioscience) and PE-Cy7-conjugated anti-CD25 (clone BC96, eBioscience). Then, cells were fixed and permeabilized using FOXP3 staining buffer set (eBioscience), according to the manufacturer’s instructions. Cells were then stained with APC-conjugated anti-FOXP3 (clone PCH101, eBiosciences) and acquired on a FACS Aria III (BD Biosciences) running FACS Diva version 8 software (Becton Dickinson). Data were analyzed using Kaluza version 1.3 (Beckman Coulter).

T cell differentiation was determined according to the expression of CD45RA and CCR7 as naive (TN, CD45RA + CCR7 +), central memory (TCM, CD45RA−CCR7 +) and effector memory (TEM, CD45RA−CCR7- and CD45RA + CCR7−).

Regulatory T cells were defined as CD25 + CD127-FOXP3 + CD4 + T cells. Further analysis of regulatory T cell subpopulations was based on the expression of FOXP3 and CD45RA as resting (rTreg, FOXP3low + CD45RA +), activated (aTreg, FOXP3high CD45RA−) and non-suppressive (nsTregFOXP3lowCD45RA−) regulatory T cells.

Induction of activated T cells was calculated as the difference between the percentage of CD25 + CD127 + T cells in GAD65-stimulated cultures and the percentage of CD25 + CD127 + T cells in medium alone.

### Clinical evaluation

In addition to C-peptide measurements during 2-h mixed meal tolerance test [25], we also used insulin dose U/kg body weight, 24 h as well as HbA1c. Insulin dose and HbA1c were combined to calculate insulin dose-adjusted A1c according to Mortensen et al. [23] whose results suggested the definition of an insulin dose-adjusted A1C (IDAA1C) as A1C (percent) + [4 × insulin dose (units per kilogram per 24 h)]. A calculated IDAA1C ≤ 9 corresponding to a predicted stimulated C-peptide > 300 pmol/l was used to define partial remission. To investigate immune response in relation to C-peptide preservation, patients were stratified into good responders (GR, *n* = 8; no loss of fasting C-peptide and loss of C-peptide AUC < 30%) and poor responders (PR, *n* = 4; decreasing fasting C-peptide and loss of C-peptide AUC ≥ 30%) according to C-peptide preservation and clinical response at 15 months, with cutoff for AUC 55% at 30 months.

### Statistical analysis

Variables that followed a normal distribution (clinical data) were presented as mean. Differences between groups were calculated using Student t-test, and differences within groups were calculated by paired t test. Variables that follow a non-normal distribution (immune data), were presented as median and nonparametric tests were applied. Differences between groups were calculated using Mann–Whitney test, and for the calculation of differences within groups, Wilcoxon test was applied. A probability level of < 0.05 was considered statistically significant. Calculations were performed using GraphPad Prism 8.0.1 for Windows (GraphPad Software, La Jolla, CA, USA).

## Results

### Clinical response at 30 months

**Fig. 1:**
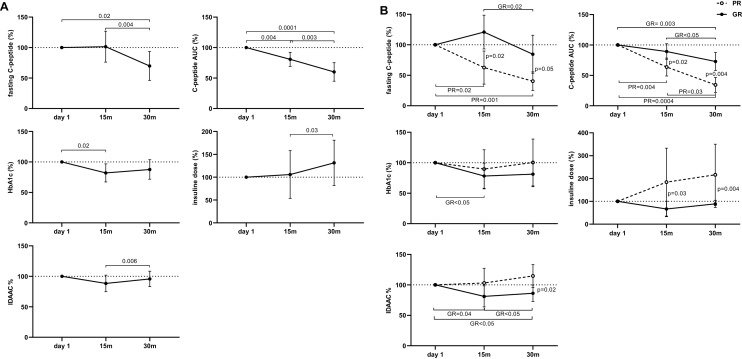
**A** Change from baseline to 30 months of fasting and stimulated C-peptide (AUC), HbA1c, IDAAC and insulin dose in type 1 diabetes patients (*n* = 12) receiving GAD-alum injections into the lymph node. Values are expressed as mean percentage. Error bars indicate 95% CI. Differences between time points were determined by paired t-Test. **B** Change from baseline of fasting and stimulated C-peptide (AUC), HbA1c, IDAAC and insulin dose in the patients stratified into good responders (GR, *n* = 8) and poor responders (PR, *n* = 4) according to their C-peptide preservation at 30 months. Error bars indicate 95% CI. Wilcoxon test was applied to calculate differences between time points and Mann–Whitney test to calculate differences between groups

Fasting and stimulated C-peptide, measured as the area under the curve (AUC), decreased after 30 months compared to both baseline and 15-month levels. Insulin requirement was higher than at 15 months, while HbA1c remained stable. IDAA1c decreased compared to 15 months, but did not differ from baseline (Fig. 1A).

Fasting and stimulated C-peptide (AUC) were significantly higher in GR patients (*n* = 8), who also had lower insulin requirement and IDAA1c compared to PR patients (*n* = 4) (Fig. 1B, Supplementary Table S1).

### Immune responses at 30 months

**Fig. 2:**
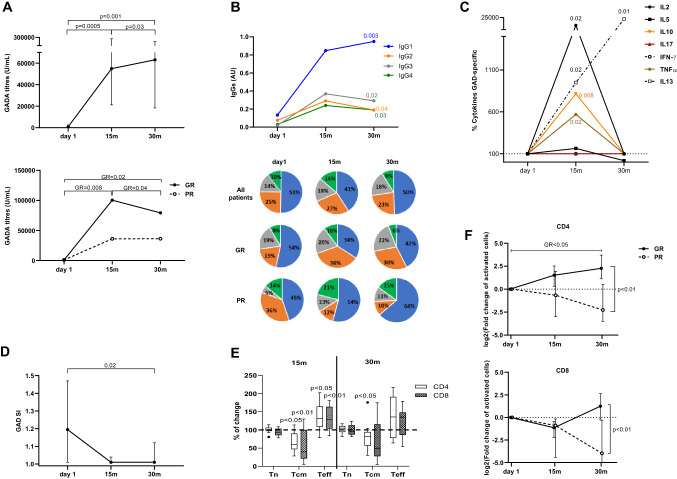
Immune response from baseline (day 1) to 30 months in type 1 diabetes patients receiving GAD-alum injections into the lymph node. **A** Median values of GADA titers (U/ml) in all patients (*n* = 12) and in patients stratified into good responders (GR, *n* = 8) and poor responders (PR, *n* = 4). **B** Median levels of IgG1, IgG2, IgG3 and IgG4 GADA subclasses (AUs), and GADA IgG subclass relative distribution at baseline, 15 and 30 months in all patients (*n* = 12) and in patients stratified into GR (*n* = 8) and PR (*n* = 4). Frequencies of each subclass were calculated with respect to the combined sum of the AUs of the four subclasses in each sample. **C** Median change of cytokine secretion from baseline. Cytokines were detected by Luminex in PBMCs supernatants after 7-day culture in the presence of GAD_65_ (5 µg/ml) or medium alone. GAD_65_-induced cytokine secretion levels are given after subtraction of spontaneous secretion from each individual and expressed as pg/mL. **D** Median values of PBMCs proliferative responses to GAD_65_. Cells were culture for 3 days with GAD_65_ (5 µg/mL) or medium, and thereafter, cells were pulsed with [^3^H] thymidine and harvested. Proliferation is expressed as stimulation index (SI) and calculated from the mean of triplicates in the presence of stimulus divided by the mean of triplicates with medium alone. SI scale started from 0 after subtracting unstimulated value index (SI unstimulated = 1). **E)** Median percentage of naïve (T_N_, CD45RA^+^CCR7^+^), central memory (T_CM_, CD45RA^−^CCR7^+^) and effector memory (T_EM_, CD45RA^−^CCR7^−^ and CD45RA^+^CCR7^−^) within CD4^+^ (white) and CD8^+^ (gray) T cells. **F** median percentages of GAD_65-_CD4^+^ and CD8^+^T cells after stimulation (5 µg/ml) in patients stratified into GR and PR. Differences within the group were calculated using Wilcoxon paired test. Error bars indicate interquartile range

GADA levels increased, more so in GR patients, through the study and were higher at 30 months than after 15 months. Analysis of GADA IgG subclasses showed that the levels of IgG1, IgG2, IgG3 and IgG4 remained higher at 30 months than baseline. Comparison of GADA between GR and PR patients showed that the increase observed in the whole cohort was due to higher levels in GR individuals, while the same difference was not observed in PR subjects (Fig. 2A).

Distribution of IgG subclasses, calculated as frequency of each subclass with respect to the combined sum of the AUs of all subclasses in each sample, was similar at 30 months to baseline distribution in the whole group. However, stratification of the samples into GR and PR showed a greater proportion of IgG2, IgG3 and IgG4 in GR individuals, while IgG1 was the predominant subclass especially in the PR individuals (Fig. 2B).

GAD_65_-induced cytokine secretion observed at earlier visits waned after 30 months, and almost all analyzed cytokines were undetectable, with the exception of IL-13 which continued to increase at 30 months compared to both baseline and 15 months (Fig. 2C, Supplementary Fig S1). The reduction of GAD_65_-induced proliferation observed at 15 months remained stable after 30 months, and proliferation was almost undetectable in all patients (Fig. 2D).

Analysis of T cell differentiation revealed that only CD4 + Tcm cells remained significantly reduced at 30 months, while Tcm and Tem fractions of CD8 + T cells did not differ significantly between baseline and 30 months (Fig. 2E). The percentage of regulatory T cell and their subpopulations did not change throughout the study (Supplementary Fig S1). GAD_65_ stimulation did not induce a significant change in activated CD4 + T cells. However, the induction of activated CD8 + T cells increased significantly from 15 to 30 months reaching similar levels as baseline (Supplementary Fig S1). When patients were stratified into GR and PR, a significant increase in GAD_65_-induced activated CD4 + T cells from baseline to 30 months was observed in the GR group (Fig. 2F). Comparison of GAD_65_-induced activated cells between the groups revealed a significantly higher fold change from baseline to 30 months of both CD4 + and CD8 + T cells in GR patients (Fig. 2F).

### Clinical and immune responses after the late booster injection of GAD-alum (DIAGNODE Extension)

**Fig. 3:**
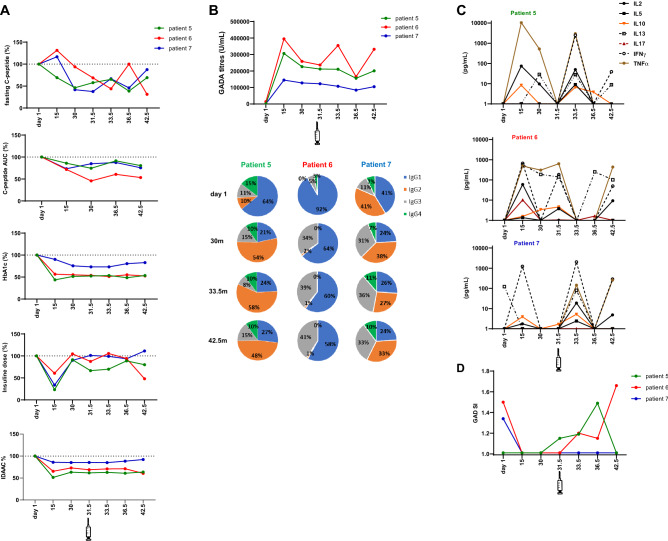
Clinical and immunological responses in patients (*n* = 3) who received a late booster injection of GAD-alum. **A** Fasting and stimulated C-peptide (AUC), HbA1c, insulin dose and IDAAC expressed as percentages. **B** GADA titers (U/ml) expressed as arbitrary units (AUs,) and relative distribution of GADA IgG subclass. Frequencies of each subclass were calculated with respect to the combined sum of the AUs of the four subclasses in sample. **C** Cytokine secretion detected by Luminex in PBMCs supernatants after 7-day culture in the presence of GAD_65_ (5 µg/ml) or medium alone. GAD_65_-induced cytokine secretion levels are given after subtraction of spontaneous secretion from each individual and expressed as pg/mL **D** PBMCs proliferative responses to GAD_65_. Cells were cultured for 3 days with GAD_65_ (5 µg/mL) or medium, and thereafter, cells were pulsed with [^3^H] thymidine and harvested. Proliferation is expressed as stimulation index (SI) and calculated from the mean of triplicates in the presence of stimulus divided by the mean of triplicates with medium alone. SI scale started from 1 after dividing by the unstimulated value index (SI unstimulated = 1). Patient 5 (green line), patient 6 (red line) and patient 7 (blue line). Statistical analysis was not applied since individual values are shown

Administration of a fourth injection of GAD-alum as a late booster dose was safe, and no adverse events were observed. Follow-up of the patients 6 and 12 months after the start of DIAGNODE extension study showed that C-peptide secretion seemed to increase in all three patients after 6 months and then remained at a stable level at 12-month follow-up, that is, still 42.5 months from the start of DIAGNODE-1. HbA1c, insulin requirement and IDAA1c seemed to stabilize after the late booster dose (Fig. 3A, Supplementary Table S2).

Analysis of the individual immunological responses showed that GADA did not further increase in subjects 5 and 7, while it was enhanced in patient 6. GADA subclass distribution seemed to be unaffected in the three individuals (Fig. 3B). Secretion of GAD_65_-induced cytokines was boosted in subjects 5 and 7, but the same clear increase was not observed in subject 6 (Fig. 3C, Supplementary Fig S2). GAD_65_-induced proliferation waned in patients 5 and 7, but increased in patient 6 (Fig. 3D). Analysis of T cell activation by GAD_65_ stimulation revealed that all three individuals reduced the activation of CD8 + T cells after the booster injection, although patient 7 maintained higher levels than baseline at the start of DIAGNODE-1 (Supplementary Fig S2).

## Conclusions

Follow-up of the patients in DIAGNODE-1 showed that C-peptide preservation observed after 6 and 15 months [21] had declined at 30 months as a mean for the total group, but remained good in 8/12 patients (good responders). Insulin requirement had increased while HbAc1 remained stable. IDAA1c decreased compared to 15 months, but was still 30 months after the treatment as good as at baseline, when the patients had less than 6-month duration of type 1 diabetes. The gradual decrease in the clinical efficacy was accompanied by a decrease in the immune response. Thus, GAD_65_-specific immune responses induced by the treatment had weaned, and most changes observed at 6 and 15 months [19–21] were undetectable. An important exception was the increased GAD_65_-induced secretion of IL-13, a main Th-2 effector cytokine well known for exerting anti-inflammatory activity, stimulate T helper cell differentiation and antibody isotype switching in B cells [29].

It was interesting that those with best C-peptide preservation, so-called good responders (GR), had higher GADA titers and higher proportion of IgG2, IgG3 and IgG4. We also observed a sustained re-call response to GAD_65_ in both CD4 + and CD8 + cells in the GR individuals, suggesting that the specific immunomodulation induced by the treatment was still persistent. Difference in the immune response at 30 months between GR and poor responders (PR) was in line with our previous findings at 15 months [21]. The increasing consensus on the heterogeneity of type 1 diabetes brings focus to the matter that, as in many other autoimmune diseases, some patients benefit from the treatments, while others do not [30, 31]. In a large meta-analyses of all patients treated with sc injections of GAD-alum, we have earlier seen the heterogeneity of the effect related to HLA type, with better response in patients with HLA DR3DQ2 [22]. Similar association was confirmed in the DIAGNODE-2 trial [24]. The small size of the group included in this pilot trial precluded the analysis of HLA-associated clinical and immunological response. Still, the three individuals, who received a fourth intra-lymphatic injection at 31,5 months from baseline and increased in C-peptide, were all HLA DR3DQ2 positive, which might support the notion that carriers of this haplotype benefit clinically from GAD-alum booster injections. Here we also show that in addition to heterogeneity of the response based on HLA types, different immune response seems to be related to C-peptide preservation.

Even though we find C-peptide preservation and clinical response still after 30 months in many patients, the efficacy as well as the immune response has declined. Then the question arises whether the immune response and the C-peptide preservation can be enhanced by giving further booster dose of the autoantigen. In the DIAGNODE Extension study certainly, only three patients were included for safety reasons, being the first-in-human approach of administration of intra-lymphatic autoantigen with a booster dose given long time after the initial treatment (in this case 2.5 years). The small number of patients is of course a limitation, but it has to be considered that this is the first-in-human trial when autoantigen has been given in a booster dose. Afterward, it is easy to conclude that the treatment worked, and therefore, more patients could have been treated, but when the study was planned the consequences were completely unknown, and could have been deleterious. However, follow-up of these patients showed that administration of the booster dose was safe and tolerable with no adverse events. Furthermore, C-peptide secretion (AUC) seemed to increase almost 4 years after the initial immune intervention, which to our knowledge has never been shown before with any type of immune intervention. HbA1c, insulin requirement and IDAAC remained stable. The clinical results were accompanied by activation of the immune response as several cytokines were induced as part of immune response to GAD_65_. The results of this small DIAGNODE Extension trial will be the basis for coming studies when booster doses will be used in autoantigen treatment of type 1 diabetes and probably also other autoimmune diseases.

Development of treatment based on the administration of antigens directly into the lymph nodes is in an early stage [32]. Results from our first trial in humans with type 1 diabetes shows that this form of treatment seems to be a promising alternative for the disease [19–21], and these results have later been confirmed in the Phase II trial DIAGNODE-2 (24). Our present results show some declining efficacy after 30 months, but the DIAGNODE Extension study suggests that repeated booster administration of GAD-alum might be a way to maintain the immunological and clinical effect. The efficacy of intra-lymphatic GAD-alum might be improved or at least maintained long term with repeated booster injections. These results are important and should be studied further.

## Supplementary Information

Below is the link to the electronic supplementary material.

Supplementary file1 (PPTX 126 KB)

Supplementary file2 (PPTX 100 KB)

Supplementary file3 (DOCX 35 KB)

## Data Availability

Data may be available from the corresponding author on reasonable request after ethical approval.

## Abbreviations

GADA: Glutamic acid decarboxylase autoantibodies
GAD-alum: Glutamic acid decarboxylase formulated with aluminum hydroxide
IDAA1c: Insulin dose-adjusted glycated hemoglobin A1c
LN: Lymph node
MMTT: Mixed meal tolerance test
PBMC: Peripheral blood mononuclear cell
SC: Subcutaneous
Th: T helper
GR: Good responders
PR: Poor responders
