# Supplementary material for: Intra-lymphatic administration of GAD-alum in type 1 diabetes: long-term follow-up and effect of a late booster dose (the DIAGNODE Extension trial)
Source: Acta Diabetol. 2022 Jan 31;59(5):687–96. doi: 10.1007/s00592-022-01852-9 (PMC8995247; doi:10.1007/s00592-022-01852-9)
Supplement: Supplementary file 1 — Supplementary file1 (PPTX 126 KB) [file 592_2022_1852_MOESM1_ESM.pptx]

## Slide 1
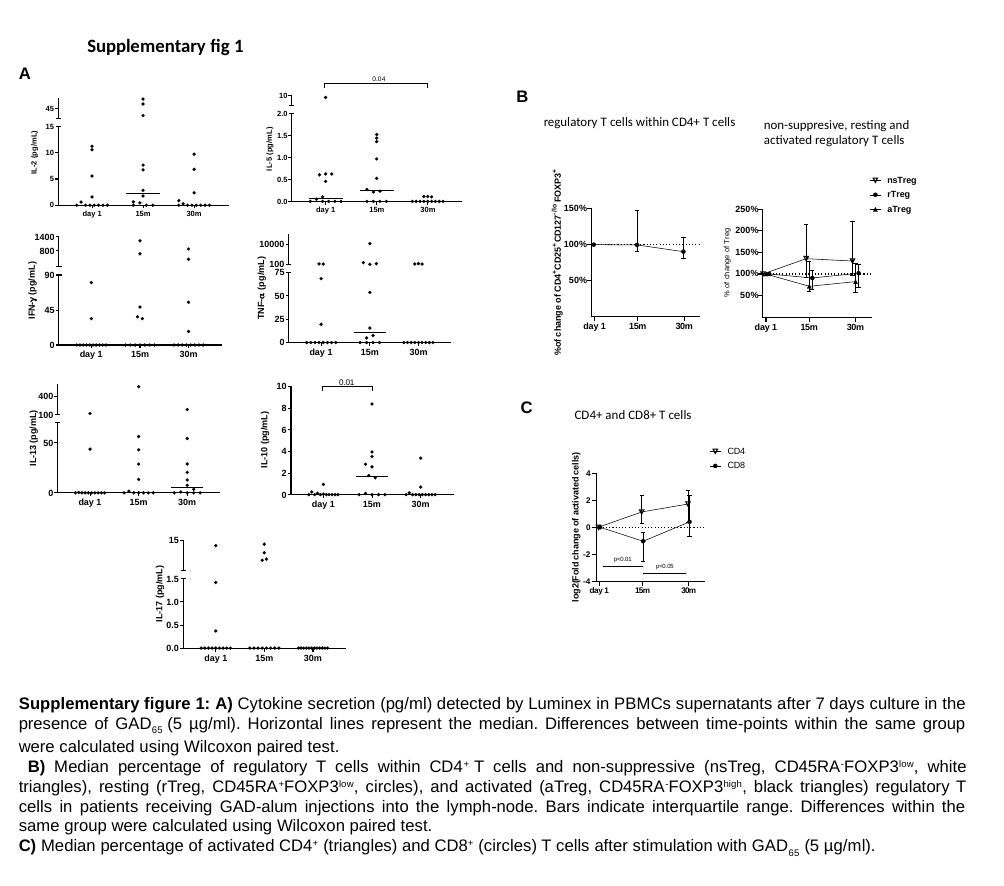

Supplementary fig 1
A
B
regulatory T cells within CD4+ T cells
non-suppresive, resting and activated regulatory T cells
C
CD4+ and CD8+ T cells
Supplementary figure 1: A) Cytokine secretion (pg/ml) detected by Luminex in PBMCs supernatants after 7 days culture in the presence of GAD65 (5 µg/ml). Horizontal lines represent the median. Differences between time-points within the same group were calculated using Wilcoxon paired test.
 B) Median percentage of regulatory T cells within CD4+ T cells and non-suppressive (nsTreg, CD45RA-FOXP3low, white triangles), resting (rTreg, CD45RA+FOXP3low, circles), and activated (aTreg, CD45RA-FOXP3high, black triangles) regulatory T cells in patients receiving GAD-alum injections into the lymph-node. Bars indicate interquartile range. Differences within the same group were calculated using Wilcoxon paired test.
C) Median percentage of activated CD4+ (triangles) and CD8+ (circles) T cells after stimulation with GAD65 (5 µg/ml).
