# Supplementary material for: Intra-lymphatic administration of GAD-alum in type 1 diabetes: long-term follow-up and effect of a late booster dose (the DIAGNODE Extension trial)
Source: Acta Diabetol. 2022 Jan 31;59(5):687–96. doi: 10.1007/s00592-022-01852-9 (PMC8995247; doi:10.1007/s00592-022-01852-9)
Supplement: Supplementary file 2 — Supplementary file2 (PPTX 100 KB) [file 592_2022_1852_MOESM2_ESM.pptx]

## Slide 1
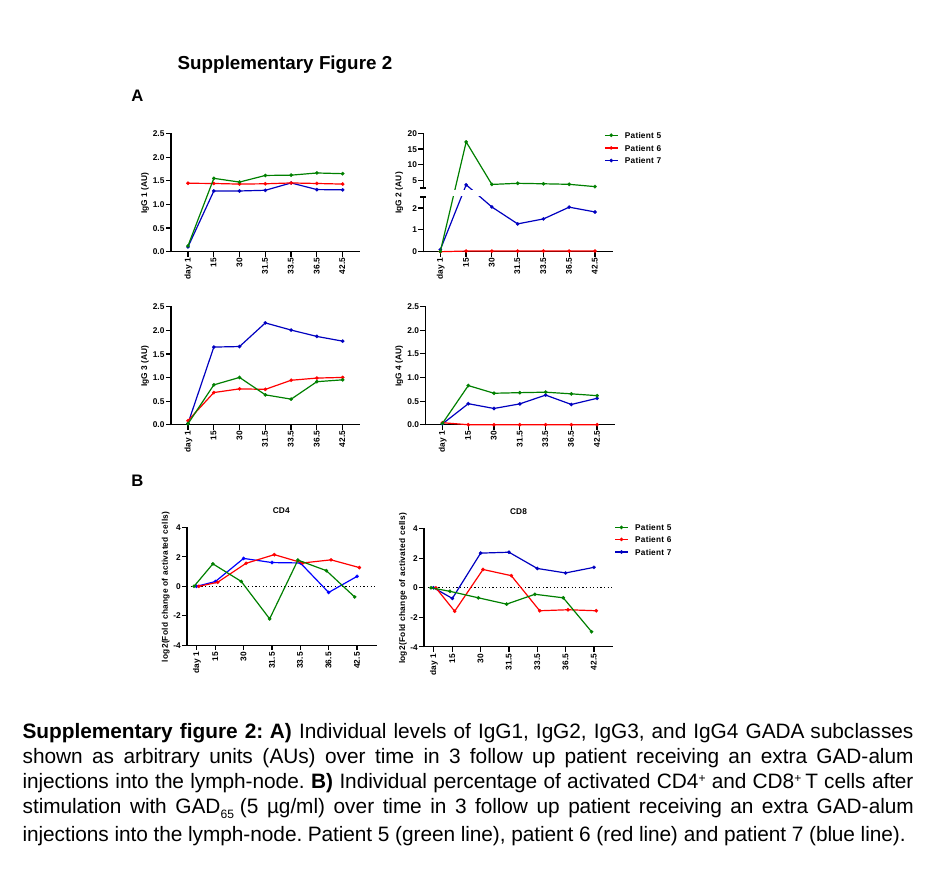

Supplementary Figure 2
A
B
Supplementary figure 2: A) Individual levels of IgG1, IgG2, IgG3, and IgG4 GADA subclasses shown as arbitrary units (AUs) over time in 3 follow up patient receiving an extra GAD-alum injections into the lymph-node. B) Individual percentage of activated CD4+ and CD8+ T cells after stimulation with GAD65 (5 µg/ml) over time in 3 follow up patient receiving an extra GAD-alum injections into the lymph-node. Patient 5 (green line), patient 6 (red line) and patient 7 (blue line).
