# Supplementary material for: Intra-lymphatic administration of GAD-alum in type 1 diabetes: long-term follow-up and effect of a late booster dose (the DIAGNODE Extension trial)
Source: Acta Diabetol. 2022 Jan 31;59(5):687–96. doi: 10.1007/s00592-022-01852-9 (PMC8995247; doi:10.1007/s00592-022-01852-9)
Supplement: Supplementary file 3 — Supplementary file3 (DOCX 35 KB) [file 592_2022_1852_MOESM3_ESM.docx]

**Supplementary Table S1:** Baseline (day 1), 15 and 30 months clinical parameters in the 12 patients who received GAD-alum injections into the lymph node (LN).

|  |  |  |  | **day 1** | | | | | | **15 months** | | | | | | **30 months** | | | | | | **Δ variable change (%) (30m-day 1)** | | | | | **Δ variable change (%) (30m-15m)** | | | | |
| --- | --- | --- | --- | --- | --- | --- | --- | --- | --- | --- | --- | --- | --- | --- | --- | --- | --- | --- | --- | --- | --- | --- | --- | --- | --- | --- | --- | --- | --- | --- | --- |
| **Patient** | **Gender** | **Age** | **Responders** | **Fast C-pep** | **C-pep AUC** | **Insulin dose** | **HbA1c** | **HbA1c (%)** | **IDAAC** | **Fast C-pep** | **C-pep AUC** | **Insulin dose** | **HbA1c** | **HbA1c (%)** | **IDAAC** | **Fast C-pep** | **C-pep AUC** | **Insulin dose** | **HbA1c** | **HbA1c (%)** | **IDAAC** | **Fast C-pep** | **C-pep AUC** | **Insulin dose** | **HbA1c** | **IDAAC** | **Fast C-pep** | **C-pep AUC** | **Insulin dose** | **HbA1c** | **IDAAC** |
| **1** | **F** | **22** | **GOOD** | 0.17 | 0.36 | 0.46 | 52 | 6.9 | 8.76 | 0.24 | 0.39 | 0.54 | 41 | 5.9 | 8.05 | 0.15 | 0.26 | 0.48 | 44 | 6.2 | 8.12 | -12 | -30 | 5 | -15 | -7 | -38 | -35 | -10 | 7 | 1 |
| **2** | **M** | **22** |  | 0.26 | 0.85 | 0.25 | 58 | 7.5 | 8.46 | 0.26 | 0.68 | 0.28 | 54 | 7.1 | 8.20 | 0.16 | 0.61 | 0.15 | 60 | 7.6 | 8.22 | -38 | -28 | -42 | 3 | -3 | -38 | -10 | -47 | 11 | 0 |
| **3** | **M** | **23** |  | 0.12 | 0.43 | 0.29 | 66 | 8.2 | 9.37 | 0.22 | 0.35 | 0.15 | 52 | 6.9 | 7.53 | 0.15 | 0.28 | 0.23 | 53 | 7.0 | 7.91 | 25 | -34 | -23 | -20 | -16 | -32 | -20 | 47 | 2 | 5 |
| **4** | **F** | **21** |  | 0.25 | 0.74 | 0.45 | 68 | 8.4 | 10.16 | 0.28 | 0.81 | 0.11 | 41 | 5.9 | 6.33 | 0.18 | 0.46 | 0.32 | 49 | 6.6 | 7.93 | -28 | -38 | -28 | -28 | -22 | -36 | -42 | 205 | 20 | 25 |
| **5** | **M** | **22** |  | 0.26 | 0.42 | 0.29 | 103 | 11.6 | 12.72 | 0.18 | 0.36 | 0.07 | 45 | 6.3 | 6.54 | 0.12 | 0.31 | 0.26 | 53 | 7.0 | 8.05 | -54 | -26 | -9 | -49 | -37 | -33 | -13 | 292 | 18 | 23 |
| **6** | **M** | **23** |  | 0.16 | 0.38 | 0.55 | 78 | 9.3 | 11.50 | 0.21 | 0.27 | 0.34 | 44 | 6.2 | 7.52 | 0.15 | 0.17 | 0.58 | 43 | 6.1 | 8.40 | -6 | -54 | 5 | -45 | -27 | -29 | -36 | 72 | -2 | 12 |
| **7** | **F** | **23** |  | 0.24 | 0.56 | 0.22 | 41 | 5.9 | 6.80 | 0.28 | 0.41 | 0.08 | 37 | 5.5 | 5.84 | 0.10 | 0.47 | 0.20 | 31 | 5.0 | 5.80 | -58 | -15 | -10 | -24 | -15 | -64 | 16 | 167 | -16 | -1 |
| **8** | **M** | **12** |  | 0.23 | 0.52 | 0.36 | 37 | 5.5 | 6.99 | 0.26 | 0.54 | 0.41 | 46 | 6.4 | 8.00 | 0.34 | 0.55 | 0.41 | 47 | 6.5 | 8.09 | 48 | 7 | 13 | 27 | 16 | 31 | 2 | 0 | 2 | 1 |
| **9** | **M** | **13** | **POOR** | 0.25 | 0.52 | 0.29 | 66 | 8.2 | 9.36 | 0.18 | 0.40 | 0.58 | 40 | 5.8 | 8.15 | 0.07 | 0.13 | 0.79 | 43 | 6.1 | 9.23 | -72 | -75 | 170 | -35 | -1 | -61 | -68 | 35 | 8 | 13 |
| **10** | **M** | **20** |  | 0.21 | 0.36 | 0.47 | 54 | 7.1 | 8.96 | 0.12 | 0.22 | 0.54 | 51 | 6.8 | 8.96 | 0.10 | 0.14 | 0.72 | 65 | 8.1 | 10.97 | -52 | -62 | 53 | 20 | 22 | -17 | -39 | 34 | 27 | 22 |
| **11** | **F** | **15** |  | 0.58 | 0.85 | 0.15 | 37 | 5.5 | 6.14 | 0.24 | 0.53 | 0.46 | 39 | 5.7 | 7.58 | 0.28 | 0.36 | 0.46 | 40 | 5.8 | 7.64 | -52 | -57 | 206 | 8 | 25 | 17 | -31 | -1 | 3 | 1 |
| **12** | **M** | **13** |  | 0.40 | 0.69 | 0.53 | 50 | 6.7 | 8.85 | 0.32 | 0.38 | 0.59 | 49 | 6.6 | 9.00 | 0.15 | 0.23 | 0.72 | 54 | 7.1 | 9.97 | -63 | -67 | 36 | 8 | 13 | -53 | -40 | 22 | 10 | 11 |

Clinical data are from each patient. Percentage of change (%) of C-peptide, Insulin dose and HbA1c from baseline (day 1) to 30 months, and from 15 to 30 months (Δ variable change) were calculated and displayed in the last column of the table.

F, female; M, male; Fast C-pep, Fasting C-peptide (nmol/L); C-pep AUC, C-peptide area under the curve (nmol/L); Insulin dose, U/kg of body weight/24 hours; HbA1c, Glycated hemoglobin (mmol/mol); IDAAC, Insulin-dose adjusted HbA1c.
